# Associations of CXCL12 polymorphisms with clinicopathological features in breast cancer: a case-control study

**DOI:** 10.1007/s11033-021-07047-9

**Published:** 2022-01-25

**Authors:** Shuai Lin, Yi Zheng, Meng Wang, Linghui Zhou, Yuyao Zhu, Yujiao Deng, Ying Wu, Dai Zhang, Na Li, Huafeng Kang, Zhijun Dai

**Affiliations:** 1 Department of Oncology, The Second Affiliated Hospital of Xi’an Jiaotong University, 710004 Xi’an, China; 2 Department of Breast Surgery, The First Affiliated Hospital, College of Medicine, Zhejiang University, 310003 Hangzhou, China

**Keywords:** Breast cancer, CXCL12, Polymorphism, Case-control study

## Abstract

**Background:**

Previous studies suggested that CXCL12 was involved in the development, metastasis, and invasion of breast cancer, and genetic variants were associated with the diagnosis and prognosis of patients with breast cancer. The present study was aimed to assess the relationships between CXCL12 polymorphisms (rs1801157, rs2297630, and rs2839693) and susceptibility and clinicopathological features of breast cancer.

**Methods:**

A case-control study was conducted in 434 breast cancer patients and 450 health controls. Student *t*-test and chi-square test were used to analyze the differences of age distribution and genotype frequencies between the two groups. Correlations between polymorphisms and clinical parameters were also assessed by chi-square test. The potential effects of the three polymorphisms on CXCL12 were investigated by the public database.

**Results:**

A statistical association was found between CXCL12 rs1801157 polymorphism and breast cancer risk, possibility of metastasis, and estrogen receptor status. Patients with rs2839693 C/T or C/T-T/T genotypes were more likely to be progesterone receptor-negative. However, no associations of rs2297630 polymorphism with breast cancer risk or any clinicopathological characteristics were observed. In addition, rs2297630 affected the splicing quantitative trait loci of CXCL12 in the subcutaneous fat, rs2839693 polymorphism affected the splicing quantitative trait loci of CXCL12 in the human breast mammary tissues.

**Conclusions:**

Those results indicated that CXCL12 polymorphisms might be potential diagnostic indicators, and more investigation is needed in the future.

**Supplementary Information:**

The online version contains supplementary material available at 10.1007/s11033-021-07047-9.

## Introduction

Breast cancer, one of the most common female malignancies worldwide, strongly affects the lives of millions of women. There were around 268,600 new cases and 41,760 deaths occurring in the United States in 2019 [1]. In China, breast cancer was responsible for 416,000 new cases and 117,000 deaths in 2020 [2]. Because of the rapid progress in diagnosis and treatment modalities, survival of breast cancer patients has been significantly improved. Five-year survival rates for localized and regional stage patients are 99% and 85%, respectively. However, for patients diagnosed at an advanced stage, the five-year survival rate is only 27% [3]. Due to the heterogeneity of cancer in terms of treatment response, recurrence, and propensity of metastasis, those commonly used diagnosis and treatment criteria and prognostic biomarkers are not suitable for everyone, so individualized tests and indicators are urgently needed.

In recent years, the role of immune system in cancer growth, elimination, and metastasis gains the increased attention of the majority of researchers. Degnim AC et al. found that in premalignant breast tissues, the densities of CD8 + T cells, CD11c + dendritic cells, CD20 + B cells, and CD68 + macrophages were higher than those in normal tissues, indicating the alterations in the immune cell composition [4]. Increased tumor infiltrating lymphocytes are associated with the reduced risk of death and distant recurrence in triple negative breast cancer patients, which make them significant predictors of prognosis [5]. Cytokines are the master regulators of immune cells, recruiting them from the bone marrow and blood to the tumor and polarizing their phenotypes within tumor microenvironment. As a vital member of cytokines, chemokines play an important role. Besides their chemotactic abilities, chemokines can directly regulate T cell development, priming and effector functions [6]. Based on the first cysteine residue position, chemokines are divided into two sub-families, CXC and CC, which are responsible for chemotaxis of neutrophils and monocytes and lymphocytes, respectively [7].

CXC members are involved in multiple processes, like embryogenesis, hematopoiesis, angiogenesis, inflammation, and cancer development by binding to their receptors. CXCL12, also called stromal cell-derived factor 1 (SDF1), is located on human chromosome 10q11.1 and widely expressed in almost all of the organs and multiple immune cells, fibroblasts, and epithelial cells [8]. CXCL12 exerts its function by binding to the seven-transmembrane G-protein-coupled receptor CXCR4 and CXCR7, which were expressed on a great diversity of cell types, including lymphocytes, hematopoietic stem cells, endothelial cells, epithelial cells, stromal fibroblasts, and cancer cells [9]. The interactions between CXCL12 and CXCR4 or CXCR7 comprise a biological axis that affects the growth, angiogenesis, and metastasis of cancers [10, 11]. Sigle knockout of CXCR4 or CXCR7 and co-knockout of CXCR4 and CXCR7 significantly reduced the proliferation, migration, and invasion of triple-negative breast cancer MDA-MB-231cells [12]. Overexpression of CXCR7 gene in gastric cancer SGC-7901 cells promoted cell proliferation, migration, and invasion, while the results were reversed after silencing CXCR7 gene [13]. In triple negative breast cancer patients, high cytoplasmic CXCR4 expression was related to lower distant recurrence and better recurrence-free survival, while high CXCL12 expression was associated with larger tumor size, positive lymph node metastasis, and higher pathologic stage [14]. Nano-delivery of IL10 trap and CXCL12 trap significantly reduced tumor growth and immunosuppressive cells and prolonged survival in orthotopic 4T1 triple-negative breast cancer models [15]. Elevated CXCL12 expression was also significantly related with the reduced absolute survival in patients with oesophagogastric, pancreatic, or lung cancer, but associated with the increased absolute survival in patients with breast cancer [16]. Higher CXCL12 protein expression indicated a better disease-free survival and overall survival in breast cancer patients, and had a positive relation with positive estrogen receptor (ER) status, negative human epidermal growth factor receptor (Her)-2 status, and small tumor size [17]. In addition, polymorphisms of CXCL12 were considered as factors affecting the susceptibility and prognosis to breast cancer. Previous studies showed a positive association between CXCL12 G801A polymorphism and breast cancer risk [18, 19]. However, in other studies, CXCL12 G801A polymorphism was not a risk factor for breast cancer [20, 21]. Thus, in this study, we aimed to figure out the association between three CXCL12 polymorphisms (rs1801157, rs2297630, and rs2839693) and breast cancer susceptibility in Chinese population. The relationships between CXCL12 polymorphisms and clinicopathological factors of breast cancer were also evaluated.

## Materials and methods

### Study population

Totally, 434 breast cancer patients and 450 healthy controls were enrolled for this case–control study. All patients were women with no other cancer and pathology diagnosed between 2013 and 2015 at the Second Affiliated Hospital of Xi’an Jiaotong University. Age and gender-matched healthy volunteers who came to the Second Affiliated Hospital of Xi’an Jiaotong University for physical examination were considered as controls.

### Genotyping assay

After anticoagulation treatment, blood samples were kept in the − 80℃ refrigerator for further use. Genomic DNA was extracted according to the phenol–chloroform extraction method and stored at − 20℃ [22]. Genotyping of three CXCL12 polymorphisms (rs1801157, rs2297630 and rs2839693) were conducted following the manufacturer’s instructions by a Sequenom MassARRAY RS1000. Primers were shown in Supplemental Table 1 and results were analyzed through Sequenom Typer 3.0 Software.

**Table 1:** Case–control analyses for CXCL12 polymorphisms (crude analyses)

Polymorphism	Model	Genotype	Control (%)	Case (%)	OR (95% CI)	*P*
rs1801157	Codominant	G/G	293(65.4%)	259(58.6%)	1	
		G/A	134(29.9%)	167(37.8%)	1.410 (1.063–1.869)	0.017
		A/A	21(4.7%)	16(3.6%)	0.862 (0.440–1.687)	0.665
	Dominant	G/G	293(65.4%)	259(58.6%)	1	
		G/A-A/A	155(34.6%)	183(42.4%)	1.336 (1.018–1.752)	0.037
	Recessive	G/G-G/A	427(95.3%)	426(96.4%)	1	
		A/A	21(4.7%)	16(3.6%)	0.764 (0.393–1.484)	0.426
	Log-additive	–	–	–	1.192 (0.947–1.501)	0.135
rs2297630	Codominant	G/G	330(73.5%)	325(73.7%)	1	
		G/A	108(24.1%)	107(24.3%)	1.01 (0.74–1.37)	0.97
		A/A	11(2.5%)	9(2.0%)	0.83 (0.34–2.03)	0.68
	Dominant	G/G	330(73.5%)	325(73.7%)	1	
		G/A-A/A	119(26.5%)	116(26.3%)	0.99 (0.73–1.33)	0.95
	Recessive	G/G-G/A	438(97.5%)	432(98.0%)	1	
		A/A	11(2.5%)	9(2.0%)	0.83 (0.34–2.02)	0.68
	Log-additive	–	–	–	0.98 (0.75–1.27)	0.86
rs2839693	Codominant	C/C	316(70.4%)	321(73.0%)	1	
		C/T	127(28.3%)	110(25.0%)	0.85 (0.63–1.15)	0.30
		T/T	6(1.3%)	9(2.0%)	1.48 (0.52–4.20)	0.46
	Dominant	C/C	316(70.4%)	321(73%)	1	
		C/T-T/T	133(29.6%)	119(27%)	0.88 (0.66–1.18)	0.39
	Recessive	C/C–C/T	443(98.7%)	431(98.0%)	1	
		TT	6(1.3%)	9(2.0%)	1.54 (0.54–4.37)	0.42
	Log-additive	–	–	–	0.93 (0.71–1.21)	0.57

*OR* odds ratio, *CI* confidence interval

### Statistical analyses

IBM SPSS Statistics Software Program Version 20 (SPSS Inc., Chicago, IL, USA) was utilized to analyze the data. Data were displayed as mean and percentage. Continuous and discrete data were analyzed by the student *t*-test and chi-square test, respectively [23]. Association between polymorphisms and clinical parameters (body mass index, menstrual status, tumor size, metastasis, disease stage, ER, progesterone receptor [PR], and Her-2 status) were assessed by chi-square test based on codominant, dominant, recessive, and log-additive models via calculating odds ratios (ORs) and 95% confidence intervals (CIs). Results were adjusted to exclude the influence of age. All statistics were two-sided, and *p* < 0.05 was considered statistically significant.

### Genotype–phenotype correlation analysis

Expression quantitative trait loci (eQTL) is a region of the genome containing DNA sequence variations that affect the expression levels of one or more genes [24]. Analysis of splicing quantitative trait loci (sQTLs) is used to assess the impact on splicing regulation. We further investigated the potential effects of three polymorphisms (rs1801157, rs2297630, and rs2839693) on CXCL12 by the public database GTEx portal (https://www.gtexportal.org/) [25].

## Results

### Characteristics of participants

There were 202 and 180 people with age ≤ 49 years in the health control and breast cancer group, respectively. There is no statistical difference in age between controls and patients with breast cancer (*p* = 0.306). Patients with normal body mass index (BMI), menstrual status, and small tumor size (≤ 2) accounted for 57.1%, 63.8%, and 47.5% in the breast cancer group, respectively. Other clinicopathological features were listed in Supplemental Table 2.

### Case–control study for CXCL12 polymorphisms

As displayed in Table 1, people carrying rs1801157 C/T or C/T-T/T genotype were more likely to get breast cancer when compared with those carrying C/C genotype (C/T *vs*. C/C: *p* = 0.017, C/T-T/T *vs*. C/C: *p* = 0.037). However, rs1801157 TT genotype carriers and CC or C/C–C/T genotype carriers had similar distributions in both groups (*p* = 0.665 and 0.426, respectively). The adjusted results were consistent with the previous ones (Table 2).

**Table 2:** Case–control analyses for CXCL12 polymorphisms (adjusted by age)

Polymorphism	Model	Genotype	Control (%)	Case (%)	OR (95%CI)	*p*
rs1801157	Codominant	G/G	293(65.4%)	259(58.6%)	1	
		G/A	134(29.9%)	167(37.8%)	1.426 (1.074–1.89)	0.014
		A/A	21(4.7%)	16(3.6%)	0.847 (0.432–1.660)	0.629
	Dominant	G/G	293(65.4%)	259(58.6%)	1	
		G/A-A/A	155(34.6%)	183(42.4%)	1.347 (1.025–1.768)	0.032
	Recessive	G/G-G/A	427(95.3%)	426(96.4%)	1	
		A/A	21(4.7%)	16(3.6%)	0.748 (0.384–1.455)	0.392
	Log-additive	–	–	–	1.196 (0.949–1.507)	0.129
rs2297630	Codominant	G/G	330(73.5%)	325(73.7%)	1	
		G/A	108(24.1%)	107(24.3%)	1.00 (0.74–1.37)	0.98
		A/A	11(2.5%)	9(2.0%)	0.80 (0.33–1.97)	0.63
	Dominant	G/G	330(73.5%)	325(73.7%)	1	
		G/A-A/A	119(26.5%)	116(26.3%)	0.98 (0.73–1.33)	0.92
	Recessive	G/G-G/A	438(97.5%)	432(98.0%)	1	
		A/A	11(2.5%)	9(2.0%)	0.80 (0.33–1.96)	0.63
	Log-additive	–	–	–	0.97 (0.74–1.26)	0.82
rs2839693	Codominant	C/C	316(70.4%)	321(73.0%)	1	
		C/T	127(28.3%)	110(25.0%)	0.86 (0.63–1.15)	0.31
		T/T	6(1.3%)	9(2.0%)	1.42 (0.50–4.05)	0.51
	Dominant	C/C	316(70.4%)	321(73%)	1	
		C/T-T/T	133(29.6%)	119(27%)	0.88 (0.66–1.18)	0.40
	Recessive	C/C–C/T	443(98.7%)	431(98.0%)	1	
		TT	6(1.3%)	9(2.0%)	1.48 (0.52–4.21)	0.46
	Log-additive	–	–	–	0.92 (0.71–1.21)	0.56

*OR* odds ratio, *CI* confidence interval

Association analyses did not indicate any significant association between rs2297630 and rs2839693 variants and breast cancer susceptibility based on the four models with all *p* > 0.5 (Table 1). After excluding the influence of age, the results did not seem to change (Table 2).

### CXCL12 polymorphisms and clinicopathological parameters

Correlations between polymorphism genotypes and BMI, menstrual status, tumor size, metastasis, disease stage, ER, PR, or Her-2 features were also assessed in the patients. It was found a negative association between rs1801157 genotypes and age and a positive relationship between rs1801157 genotypes and disease stage or ER status (Table 3). No meaningful correlations were found between rs2297630 polymorphism and any clinicopathological features (Table 4). In addition, compared with patients with rs2839693 C/C genotype, patients carrying C/T and C/T-T/T genotypes were more likely to be PR-negative (Table 5, C/T *vs*. C/C: *p* = 0.046, C/T-T/T *vs*. C/C: *p* = 0.023).

**Table 3:** Association between rs1801157 polymorphism and clinicopathological parameters

rs1801157	G/G	G/A	A/A	G/A + A/A
Age				
> 49/≤49	156/91	85/78	11/5	96/83
OR (95% CI)	1.00 (references)	0.64(0.42–0.95)	1.28(0.45–4.18)	0.67(0.46–1.00)
* p*		0.027*	0.653	0.049*
BMI (kg/m^2^)				
≥ 23/ < 23	88/159	71/92	7/9	78/101
OR (95% CI)	1.00 (references)	1.39(0.93–2.09)	1.41(0.49–3.90)	1.40(0.94–2.07)
* P*		0.107	0.514	0.097
Menstrual status				
Yes/no	163/84	97/66	12/4	109/70
OR (95% CI)	1.00 (references)	0.76(0.50–1.14)	1.55(0.52–5.66)	0.80(0.54–1.20)
* p*		0.183	0.462	0.28
Tumor size (cm)				
> 2/≤ 2	126/121	90/73	8/8	98/81
OR (95% CI)	1.00 (references)	1.18(0.80–1.76)	0.96(0.34–2.69)	1.16(0.79–1.71)
* p*		0.404	0.937	0.446
Metastasis				
Positive/negative	122/125	96/67	4/12	100/79
OR (95% CI)	1.00 (references)	1.47(0.99–2.19)	0.34(0.09–1.01)	1.30(0.88–1.91)
* p*		0.06	0.069	0.187
TNM stage				
III-IV/I-II	62/185	60/103	4/12	64/115
OR (95% CI)	1.00 (references)	1.74(1.13–2.67)	0.99(0.27–2.97)	1.66(1.09–2.53)
* p*		0.012*	0.992	0.018
ER				
Positive/negative	155/92	120/43	13/3	153/46
OR (95% CI)	1.00 (references)	1.66(1.08–2.57)	2.57(0.80–11.4)	1.72(1.13–2.63)
* p*		0.023*	0. 149	0.012*
PR				
Positive/negative	136/111	97/66	9/7	106/73
OR (95% CI)	1.00 (references)	1.20(0.80–1.79)	1.05(0.38–3.02)	1.19(0.80–1.75)
* p*		0.374	0.926	0.393
Her-2				
Positive/negative	106/41	70/93	5/11	75/104
OR (95% CI)	1.00 (references)	1.00(0.67–1.49)	0.60(0.19–1.72)	0.96(0.65–1.42)
* p*		0.995	0.364	0.834

*OR* odds ratio, *CI* confidence interval, *BMI* body mass index, *TNM* tumor mode metastasis, *ER* estrogen receptor, *PR* progesterone receptor, *Her* human epidermal growth factor receptor

**Table 4:** Association between 2,297,630 polymorphism and clinicopathological parameters

rs2297630	A/A	G/A	G/A + G/G
Age			
> 49/≤49	179/134	62/42	69/44
OR (95% CI)	1.00 (references)	1.11(0.71–1.74)	1.17(0.76–1.83)
* P*		0.664	0.474
BMI (kg/m^2^)			
≥23/ < 23	123/190	41/63	41/72
OR (95% CI)	1.00 (references)	1.01(0.64–1.58)	0.88(0.56–1.37)
* P*		0.982	0.573
Menstrual status			
Yes/no	200/113	65/39	71/42
OR (95% CI)	1.00 (references)	0.94(0.60–1.50)	0.96(0.60–1.50)
* p*		0.798	0.84
Tumor size (cm)			
> 2/≤2	172/141	49/55	53/60
OR (95% CI)	1.00 (references)	0.73(0.47–1.14)	0.143(0.47–1.11)
* p*		0.166	0.143
Metastasis			
Positive/negative	170/143	50/54	55/58
OR (95% CI)	1.00 (references)	0.78(0.50–1.21)	0.80(0.52–1.23)
* p*		0.27	0.304
TNM stage			
III-IV/I-II	96/217	28/76	32/81
OR (95% CI)	1.00 (references)	0.83(0.50–1.35)	0.89(0.55–1.43)
* p*		0.469	0.64
ER			
Positive/negative	211/102	71/33	76/37
OR (95% CI)	1.00 (references)	1.04(0.65–1.69)	0.99(0.63–1.58)
* p*		0.871	0. 976
PR			
Positive/negative	174/139	61/43	67/46
OR (95% CI)	1.00 (references)	1.13(0.72–1.78)	
* p*		0.585	0.497
Her-2			
Positive/negative	128/185	47/57	53/60
OR (95% CI)	1.00 (references)	1.19(0.76–1.86)	1.28(0.83–1.97)
* p*		0.442	0.269

*OR* odds ratio, *CI* confidence interval, *BMI* body mass index, *TNM* tumor mode metastasis, *ER* estrogen receptor, *PR* progesterone receptor, *Her* human epidermal growth factor receptor

**Table 5:** Association between rs2839693 polymorphism and clinicopathological parameters

rs2839693	C/C	C/T	T/T	C/T + T/T
Age				
> 49/≤ 49	180/129	63/43	6/3	69/46
OR (95% CI)	1.00 (references)	1.05(0.67–0.831)	1.43(0.37–6.89)	1.08(0.70–1.67)
* p*		0.831	0.615	0.745
BMI (kg/m^2^)				
≥ 23/ < 23	117/192	41/65	4/5	78/101
OR (95% CI)	1.00 (references)	1.04(0.65–1.62)	1.31(0.32–5.06)	1.05(0.68–1.63)
* p*		0.881	0.689	0.811
Menstrual status				
Yes/no	197/112	66/40	8/1	74/41
OR (95% CI)	1.00 (references)	0.94(0.60–1.49)	4.55(0.82–84.92)	1.03(0.66–1.61)
* p*		0.784	0.156	0.91
Tumor size (cm)				
> 2/≤ 2	157/152	63/43	5/4	68/47
OR (95% CI)	1.00 (references)	1.42(0.91–2.23)	1.21(0.31–4.97)	1.40(0. 91–2.17)
* p*		0.126	0.779	0.128
Metastasis				
Positive/negative	161/148	57/49	5/4	68/47
OR (95% CI)	1.00 (references)	1.07(0.69–1.67)	1.15(0.30–4.97)	1.40(0.91–2.17)
* p*		0.766	0.838	0.187
TNM Stage				
III-IV/I-II	90/219	34/72	2/7	36/79
OR (95% CI)	1.00 (references)	1.15(0.71–1.84)	0.70(0.10–2.94)	1.11(0.69–1.76)
* p*		0.567	0.654	0.663
ER				
Positive/negative	214/95	66/40	6/3	72/43
OR (95% CI)	1.00 (references)	0.73(0.46–1.17)	0.89(0.23–4.28)	0.74(0.48–1.17)
* p*		0.186	0. 868	0.195
PR				
Positive/negative	186/123	52/44	3/6	55/60
OR (95% CI)	1.00 (references)	0.64(0.41–0.99)	0.33(0.07–1.27)	0.61(0.39–0.93)
* p*		0.046*	0.122	0.023*
Her-2				
Positive/negative	133/176	46/60	2/7	48/67
OR (95% CI)	1.00 (references)	1.01(0.65–1.58)	0.38(0.06–1.59)	0.95(0.61–1.46)
* p*		0.949	0.23	0.809

*OR* odds ratio, *CI* confidence interval, *BMI* body mass index, *TNM* tumor mode metastasis, *ER* estrogen receptor, *PR* progesterone receptor, *Her* human epidermal growth factor receptor

### The results of genotype–phenotype correlation analysis

**Fig. 1:**
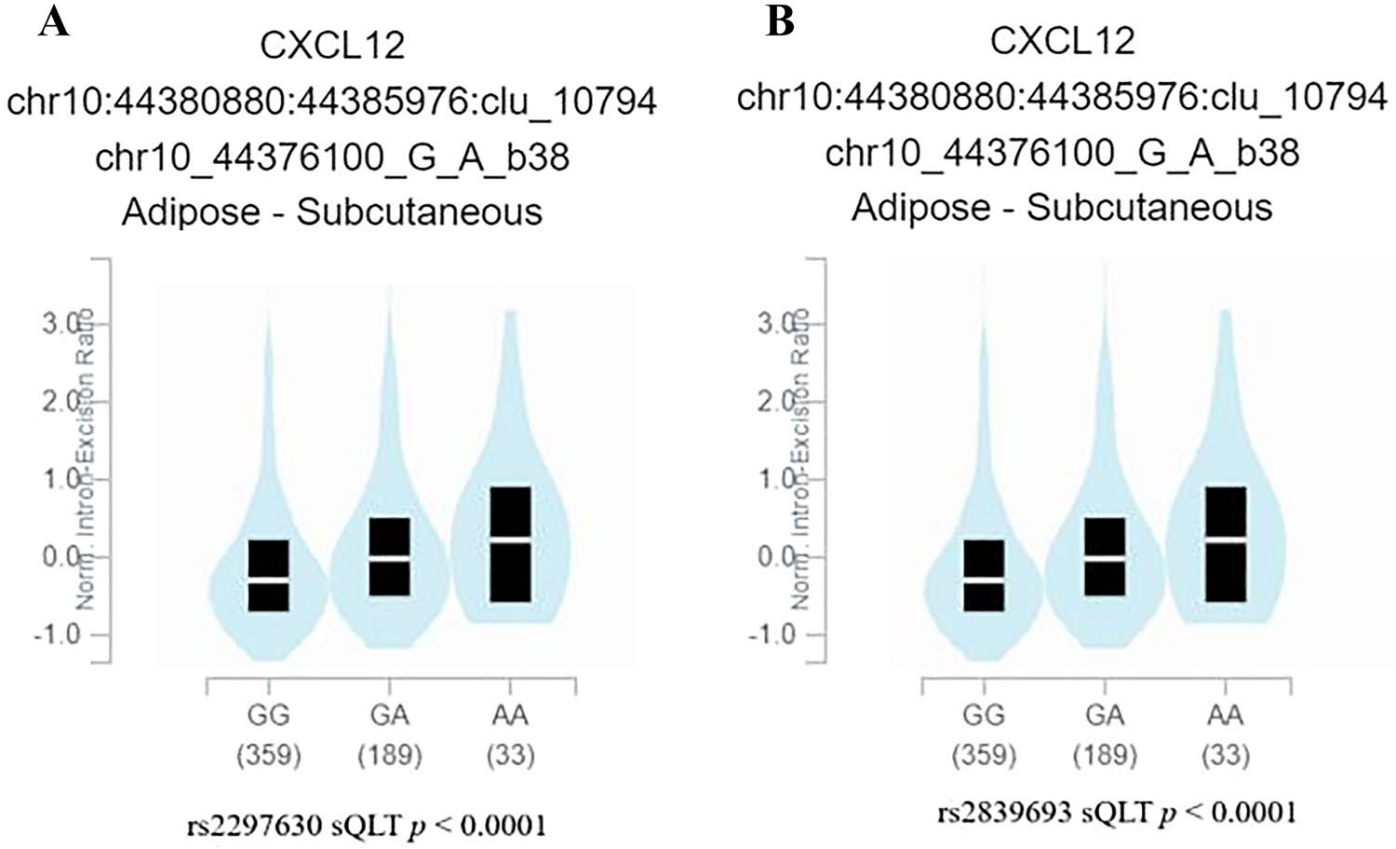
Genotype–phenotype correlation analysis rs2297630 and rs2839693 in the CXCL12 gene. Shown is the sQTL analysis for the **A** rs2297630 in in subcutaneous fat and **B** rs2839693 polymorphisms in human breast mammary tissues

To further assess the functional association of rs1801157, rs2297630, and rs2839693 and CXCL12 expression, we searched related data in public database GTEx portal. No significant eQTLs were found for SNP rs1801157, rs2297630, and rs2839693 in breast eQTL tissues of CXCL12. We found that rs2297630 affected the sQTLs of CXCL12 in the subcutaneous fat (Fig. 1A), rs2839693 polymorphism affected the sQTLs of CXCL12 in the human breast mammary tissues (Fig. 1B).

## Discussion

Published studies suggested a close association between genetic variations of CXCL12 and multiple kinds of malignant cancer. In this study, we evaluated the association between three CXCL12 polymorphisms (rs1801157, rs2297630 and rs2839693) with breast cancer. Rs1801157 polymorphism is the most studied variation with a G to A mutation at position 801 in the 3’-untranslated region in its β transcriptional splice variant of CXCL12 [26]. We found a positive relationship between CXCL12 rs1801157 polymorphism and breast cancer susceptibility. However, this relationship only occurred when compared the distribution of GA or GA-A/A genotype carriers with that of GG carriers between controls and cases. Patients with G/A genotype are more likely to process to III/IV stage and ER positive. The results of genotype–phenotype correlation analysis indicated that rs1801157, rs2297630 and rs2839693 had no effect on the expression of CXCL12 in human breast tissues, but rs2297630 and rs2839693 may affect the splicing regulation of CXCL12. However, our results were inconsistent with the previous meta-analysis, which indicated that CXCL12 rs1801157 polymorphism increased the risk of breast cancer in allelic genetic, homozygote, heterozygote, recessive genetic and dominant genetic models [18, 27]. We speculated that the reason for this difference may be because the meta-analysis studies integrated the results of multiple researches and include more patients, while our study only included breast cancer patients from one hospital in China.

Our study firstly explored the relationship of rs2297630 and rs2839693 polymorphisms with breast cancer. Unfortunately, we found the two genetic variants had no effect on the susceptibility of breast cancer. In subgroup analyses, we observed patients with rs2839693 C/T or C/T-T/T genotypes were more PR-negative, which is clinically used to predict whether early or advanced breast cancer patients are acceptable for endocrine therapy. Both rs2297630 and 2,839,693 polymorphisms were located within intro 3 of CXCL12, only rs2297630 was reported to influence the plasma SDF-1alpha level and circulating endothelial progenitor cell number [28]. Though the association between rs2297630 and rs2839693 polymorphisms and cancer have not been reported, relationships between the two polymorphisms and some diseases had been investigated. Rs2297630 polymorphism was found to be significantly linked to type 2 diabetes mellitus, dyslipidemia, post-transplant thrombocytopenia in kidney allograft recipient, coronary artery disease and other diseases [23, 29–31]. Rs2839693 polymorphism was associated with the susceptibility to sepsis, coronary artery disease in men and childhood primary immune thrombocytopenia and might influence the outcome of patients with cardiovascular disease [31–33].

Several limitations of this case–control study should be acknowledged. First, we only recruited 434 breast cancer patients and 450 health controls, and large sample size is needed to improve the credibility of the results. Second, only three polymorphisms were investigated in this study, which did not include all single nucleotide polymorphisms (SNPs) of CXCL12. Other CXCL12 alterations and the expression level of CXCL12 gene are needed to be evaluated in the future. Finally, environmental exposure and ethnic differences may affect the susceptibility and clinical indicators of breast cancer, so the correlation between CXCL12 and other factors remains to be studied.

In conclusion, the present study suggested that CXCL12 rs1801157 was significantly related with breast cancer risk, disease stage and ER feature, while CXCL12 rs2297630 or rs2839693 had no association with breast cancer susceptibility or clinicopathological parameters, except for a negative correlation of rs2839693 with PR feature. Those results indicated CXCL12 polymorphisms might be potential diagnosis indicators and more investigation is needed in the future.

## Supplementary Information

Below is the link to the electronic supplementary material.

Supplementary file1 (DOCX 14 KB)

Supplementary file1 (DOCX 15 KB)

## Data Availability

The data analyzed are available from the corresponding authors on reasonable request.

## References

[1] Ahmad A (2019). Breast cancer statistics: recent trends. Adv Exp Med Bio.

[2] Cao W, Chen HD, Yu YW (2021). Changing profiles of cancer burden worldwide and in China: a secondary analysis of the global cancer statistics 2020. Chin Med J.

[3] Siegel RL, Miller KD, Jemal A (2018). Cancer statistics, 2018. CA Cancer J Clin.

[4] Degnim AC, Hoskin TL, Arshad M (2017). Alterations in the immune cell composition in premalignant breast tissue that precede breast cancer development. Clinical Cancer Res.

[5] Stovgaard ES, Nielsen D, Hogdall E (2018). Triple negative breast cancer - prognostic role of immune-related factors: a systematic review. Acta Oncol.

[6] Franciszkiewicz K, Boissonnas A, Boutet M (2012). Role of chemokines and chemokine receptors in shaping the effector phase of the antitumor immune response. Cancer Res.

[7] Palomino DC, Marti LC (2015). Chemokines and immunity Einstein.

[8] Shirozu M, Nakano T, Inazawa J (1995). Structure and chromosomal localization of the human stromal cell-derived factor 1 (SDF1) gene. Genomics.

[9] Meng W, Xue S, Chen Y (2018). The role of CXCL12 in tumor microenvironment. Gene.

[10] Nazari A, Khorramdelazad H, Hassanshahi G (2017). Biological/pathological functions of the CXCL12/CXCR4/CXCR7 axes in the pathogenesis of bladder cancer. Int J Clin Oncol.

[11] Teicher BA, Fricker SP (2010). CXCL12 (SDF-1)/CXCR4 pathway in cancer. Clinical Cancer Res.

[12] Yang M, Zeng C, Li P (2019). Impact of CXCR4 and CXCR7 knockout by CRISPR/Cas9 on the function of triple-negative breast cancer cells. Onco Targets Ther.

[13] Xin Q, Zhang N, Yu HB (2017). CXCR7/CXCL12 axis is involved in lymph node and liver metastasis of gastric carcinoma. World J Gastroenterol.

[14] Shim B, Jin MS, Moon JH (2018). High cytoplasmic CXCR4 expression predicts prolonged survival in triple-negative breast cancer Patients Treated with Adjuvant Chemotherapy. J Pathol Transl Med.

[15] Shen L, Li J, Liu Q (2018). Local blockade of interleukin 10 and C-X-C Motif chemokine ligand 12 with nano-delivery promotes antitumor response in murine cancers. ACS Nano.

[16] Samarendra H, Jones K, Petrinic T (2017). A meta-analysis of CXCL12 expression for cancer prognosis. Br J Cancer.

[17] Liu H, Li Z, Deng M (2018). Prognostic and clinicopathological value of CXCL12/SDF1 expression in breast cancer: A meta-analysis. Clin Chim Acta.

[18] Gong H, Tan M, Wang Y (2012). The CXCL12 G801A polymorphism and cancer risk: evidence from 17 case-control studies. Gene.

[19] Khalid S, Hanif R (2017). Association of rs1801157 single nucleotide polymorphism of CXCL12 gene in breast cancer in Pakistan and in-silico expression analysis of CXCL12-CXCR4 associated biological regulatory network. PeerJ.

[20] Kruszyna L, Lianeri M, Rubis B (2010). CXCL12-3' G801A polymorphism is not a risk factor for breast cancer. DNA Cell Biol.

[21] de Oliveira KB, Oda JM, Voltarelli JC (2009). CXCL12 rs1801157 polymorphism in patients with breast cancer, Hodgkin's lymphoma, and non-Hodgkin's lymphoma. J Clin Lab Anal.

[22] Dmitrenko OP, Karpova NS, Nurbekov MK (2020). I/D polymorphism gene ACE and risk of preeclampsia in women with gestational diabetes mellitus. Dis Markers.

[23] Choi KH, Chang Y, Shah T (2019). Analysis of genetic and clinical risk factors of post-transplant thrombocytopenia in kidney allograft recipients. Transpl Immunol.

[24] Amirian ES, Armstrong GN, Zhou R (2016). The glioma international case-control study: a report from the genetic epidemiology of glioma international consortium. Am J Epidemiol.

[25] Zhu J, Fu W, Jia W (2018). Association between ner pathway gene polymorphisms and wilms tumor risk. Mol Ther Nucleic Acids.

[26] Zhu K, Jiang B, Hu R (2014). The CXCL12 G801A polymorphism is associated with cancer risk: a meta-analysis. PLoS ONE.

[27] Shen W, Cao X, Xi L (2012). CXCL12 G801A polymorphism and breast cancer risk: a meta-analysis. Mol Biol Rep.

[28] Xiao Q, Ye S, Oberhollenzer F (2008). SDF1 gene variation is associated with circulating SDF1alpha level and endothelial progenitor cell number: the Bruneck Study. PLoS ONE.

[29] Yin Q, Sun K, Xiang X (2019). Identification of novel CXCL12 genetic polymorphisms associated with type 2 diabetes mellitus: a Chinese sib-pair study. Genet Test Mol Biomarkers.

[30] Wang A, Liu X (2018). Association of CXCL12/CXCR4 gene polymorphisms with genetic risk and severity of coronary stenosis in patients with coronary artery disease. Journal of Zhejiang University Medical sciences.

[31] Wang X, Zhang AQ, Gu W (2019). Clinical relevance of single nucleotide polymorphisms in the CXCL1 and CXCL12 genes in patients with major trauma. J Ttrauma Acute Care Surg.

[32] Zhang J, Ma H, Gao J (2017). Variants in the CXCL12 gene was associated with coronary artery disease susceptibility in Chinese Han population. Oncotarget.

[33] Rath D, Schaeffeler E, Winter S (2016). SDF1 polymorphisms influence outcome in patients with symptomatic cardiovascular disease. PLoS ONE.

