# Supplementary material for: Associations of CXCL12 polymorphisms with clinicopathological features in breast cancer: a case-control study
Source: Mol Biol Rep. 2022 Jan 25;49(3):2255–63. doi: 10.1007/s11033-021-07047-9 (PMC8863681; doi:10.1007/s11033-021-07047-9)
Supplement: Supplementary file 1 — Supplementary file1 (DOCX 14 KB) [file 11033_2021_7047_MOESM1_ESM.docx]

**Supplemental table 1. Primers used in this study**

| SNP_ID | 1st-PCRP | 2nd-PCRP | UEP_SEQ |
| --- | --- | --- | --- |
| rs1801157 | ACGTTGGATGACCCCCTTCTCCATCCACAT | ACGTTGGATGACACTGCTGCCTCAGCTCA | GGCAGAAGAGGCAGACC |
| rs2297630 | ACGTTGGATGCTGCGCCCCCTTAGATAAAA | ACGTTGGATGAGTTTAACCAGTACACGGGC | ACGGGCCAGTGTTAAAT |
| rs2839693 | ACGTTGGATGCTCTGTTGCTCTCCATTCTC | ACGTTGGATGGTCATCAAAGCATACAGAGG | GACGACAGGATGCTCTAGG |
