# Supplementary material for: Associations of CXCL12 polymorphisms with clinicopathological features in breast cancer: a case-control study
Source: Mol Biol Rep. 2022 Jan 25;49(3):2255–63. doi: 10.1007/s11033-021-07047-9 (PMC8863681; doi:10.1007/s11033-021-07047-9)
Supplement: Supplementary file 2 — Supplementary file1 (DOCX 15 KB) [file 11033_2021_7047_MOESM2_ESM.docx]

**Supplemental Table 2. Basic and clinicopathological characteristics of breast cancer patients**

| **Characteristics** | **Number of Cases (%)** |
| --- | --- |
| **Age** |  |
| $\text{≤}$ 49 | 180 (41.5) |
| > 49 | 254 (58.5) |
| **BMI** |  |
| Thin (BMI < 18.5)  Normal (18.5 $\text{≤}$ BMI ) | 20 (4.6)  248 (57.1) |
| Obesity (BMI > 25) | 68 (15.7) |
| Overweight | 98 (22.6) |
| **Menstrual-status** |  |
| no | 157 (36.2) |
| yes | 277 (63.8) |
| **Tumor size** |  |
| $\text{≤}$ 2 | 206 (47.5) |
| > 5 | 24 (5.5) |
| 2-5 | 204 (47) |
| **Metastasis** |  |
| negative | 207 (47.7) |
| positive | 227 (52.3) |
| **TNM Stage** |  |
| I | 114 (26.3) |
| II | 192 (44.2) |
| III | 89 (20.5) |
| IV | 39 (9) |
| **ER** |  |
| negative | 212 (48.8) |
| positive | 222 (51.2) |
| **PR** |  |
| negative | 142 (32.7) |
| positive | 292 (67.3) |
| **Her-2** |  |
| negative | 189 (43.5) |
| positive | 245 (56.5) |
| **Ki67** |  |
| $\text{≤}$ 14 | 250 (57.6) |
| > 14 | 184 (42.4) |

BMI, body mass index; TNM, tumor mode metastasis; ER, estrogen receptor; PR, progesterone receptor; Her, human epidermal growth factor receptor.
